# Low-Cycle Fatigue of FRP Strips Glued to a Quasi-Brittle Material

**DOI:** 10.3390/ma14247753

**Published:** 2021-12-15

**Authors:** Enzo Martinelli, Antonio Caggiano

**Affiliations:** 1 Department of Civil Engineering, University of Salerno, 84084 Fisciano, Italy; 2 Institute of Construction and Building Materials, Technical University of Darmstadt, Franziska-Braun-Straße 3, 64287 Darmstadt, Germany; caggiano@wib.tu-darmstadt.de

**Keywords:** FRP, concrete, debonding, cyclic actions

## Abstract

This paper aims at further advancing the knowledge about the cyclic behavior of FRP strips glued to quasi-brittle materials, such as concrete. The results presented herein derive from a numerical model based on concepts of based on fracture mechanics and already presented and validated by the authors in previous works. Particularly, it assumes that fracture processes leading to debonding develop in pure mode II, as is widely accepted in the literature. Starting from this assumption (and having clear both its advantages acnd shortcomings), the results of a parametric analysis are presented with the aim of investigating the role of both the mechanical properties of the interface bond–slip law and a relevant geometric quantity such as the bond length. The obtained results show the influence of the interface bond–slip law and FRP bond length on the resulting cyclic response of the FRP-to-concrete joint, the latter characterized in terms of S-N curves generally adopted in the theory of fatigue. Far from deriving a fully defined correlation among those parameters, the results indicate general trends that can be helpful to drive further investigation, both experimental and numerical in nature.

## 1. Introduction

In the fields of civil and structural engineering, the use of Fiber-Reinforced Polymers (FRPs) has become very common as a strengthening technique for underperforming or damaged existing structures [1]. In fact, several mechanical models can be found in the international literature, which allow for properly designing FRP strengthening of concrete members in either under-designed or degraded structures [2]. Furthermore, recently released codes and guidelines have further consolidated the practice of adopting these FRP systems in practical applications [3,4].

Nevertheless, some issues are still open regarding the cracking processes that result in the debonding of Externally Bonded (EB) FRP strips [5]. Although several studies, both experimental [6,7] and theoretical [8,9] in nature, have been carried out with the aim to understand the bond behavior of FRP strips glued to concrete, the main knowledge gained on that subject is restricted to the case of static loads and monotonic actions. Consequently, current code provisions do not explicitly take into account the effect of cyclic actions on the possible development of FRP-to-concrete debonding [3,4].

However, FRP strips are very often employed in the structural strengthening of RC members subjected to either seismic shaking or traffic loads, which are clearly cyclic in nature, though they generally result in a significantly different response in terms of the number of cycles leading to failure.

In spite of this, many fewer studies are currently available in the literature about FRP strips glued to quasi-brittle materials such as concrete and subjected to cyclic actions. Some of them observed the cyclic response under wide amplitude oscillations hence, the low number of cycles needed for debonding: this behavior is likely to occur under earthquake-induced actions and can be approached as a low-cycle fatigue problem [10]. Conversely, other researchers investigated the response under cycles of smaller amplitude [11,12], which is the case for FRP strips employed in strengthening bridges and other structures subjected to traffic loads. 

As a matter of fact, the available studies mainly report empirical observations from experimental tests, with few modelling developments and no consistent design-oriented formulations. 

Conversely, on the modelling side, an analytical expression was proposed for a bond–slip model aimed at simulating the response observed both in monotonic and cyclic tests executed on aramid carbon and polyacetal FRP strips glued to concrete [13]. Particularly, the bond–slip law under consideration recalls the Popovics stress–strain law for concrete in compression, which, consistent with the empirical nature of the work, needs to be calibrated experimentally. 

The influence of the strain-rate effect, albeit under monotonical actions, has been investigated by considering both a local concrete damage model [14] and the foundations of fracture mechanics including the well-known Duvaut–Lions overstress viscoplastic approach [15].

As for the cyclic behavior, the potential of simulating the resulting Cohesive Zone Models (CZM) is discussed in the literature [16] and a recent model has been proposed by following this general approach [17].

On the one hand, a coupled damage-plasticity model, based on a bilinear elastic-softening bond–slip law, has been proposed [18]. On the other hand, a model based on fracture mechanics concepts was formulated with the aim to simulate by assuming two alternative bond–slip laws (namely, linear-exponential and bilinear) [19]: the model was validated with respect to some relevant experimental results available in the literature [13,20] and the elastic-exponential bond–slip law demonstrated a slightly higher accuracy in simulating the considered experimental evidence. 

A first parametric analysis carried out by means of the model in question highlighted the importance of the role that mechanical parameters play in the resulting cyclic response. Particularly, it pointed out that, unlike in the case of monotonic loading processes, bond length controls the resulting cyclic response, also in the cases of bond lengths longer than the so-called “effective” transfer length determined for monotonic loading [21]. 

Furthermore, a numerical investigation on the influence of the shape of the assumed bond–slip law on the resulting cyclic response of FRP-to-concrete was conducted by employing the model in consideration [22], which pointed out some peculiar aspects of the cyclic response that do not follow well-established relationships determined under monotonic actions.

This paper details further developments made by exploiting the simulation capabilities of the aforementioned model. After a short summary of the main assumptions and the final equation which it is based upon, a wide parametric analysis is presented with the aim to show how the properties of the FRP strip and the other relevant parameters influence the resulting S-N curve [23], which is generally considered for describing the fatigue behavior of the considered FRP-to-concrete joints. 

Therefore, Section 2 outlines the main assumptions on which the theoretical model is based and mentions the key aspects of the numerical procedure implemented with the aim to run an incremental-iterative analysis tool. Section 3 presents the main relevant quantities and summarizes the most significant results of a parametric analysis carried out for the purpose of understanding the fatigue behavior of FRP-to-concrete joints. This section also shows the resulting S-N curves for FRP-to-concrete joints characterized by particularly meaningful combinations of the relevant engineering parameters (i.e., fracture energy of the interface, specific axial stiffness of the composite strip and bond length of the joint). As a final result, a possible correlation between the two parameters that characterize the S-N curves and the aforementioned engineering parameters was found. Finally, Section 5 states the main findings of this study and introduces potential future development of the present research line. The numerical code implemented as part of the present study is available to readers as Supplementary Materials, it can be found in Open Access on Zenodo (10.5281/zenodo.5773837).

## 2. Outline of the Numerical Model

This section outlines the main assumptions, some analytical details and the essential information about numerical implementation and validation of a theoretical model formulated for simulating the cyclic response of FRP strips glued to a quasi-brittle material (hereafter referred to as “concrete” for the sake of brevity) subjected to cyclic actions.

### 2.1. Fundamental Assumptions

The model formulation is based on the following assumptions, which are widely accepted in the scientific literature:

- The cracking (debonding) process develops in pure “mode II” throughout the FRP-to-concrete interface;
- The bond–slip law consists of a first linear-elastic branch followed by a softening one intended to simulate the effect of the debonding process;
- An exponential expression is assumed for the post-elastic softening branch of the bond–slip law;
- The concrete substrate is assumed to be behave as a rigid body.

### 2.2. Main Equations

**Figure 1:**
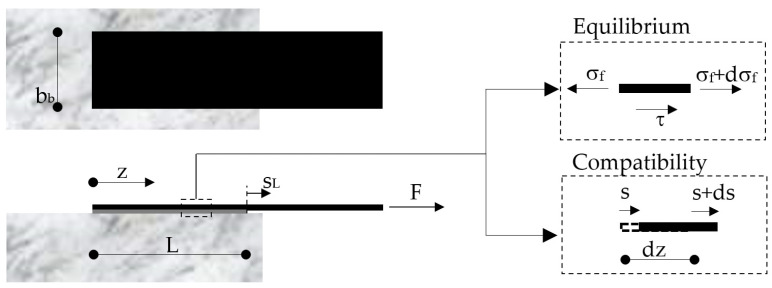
Single-lap shear test of an FRP-to-concrete bonded joint.

Figure 1 describes the FRP-to-concrete joint under consideration and provides a schematic of both the equilibrium and compatibility conditions of the generic infinitesimal segment of the FRP as resulting from the four main assumptions listed in Section 2.1.

The equilibrium condition can be mathematically expressed as follows:

(1) $$\frac{d\sigma_{f}\left(z\right)}{dz}+\frac{\tau \left(z\right)}{t_{f}}=0,$$

where *σ_f_*(*z*) is the axial stress in the transversal section of the FRP (supposed uniform across the width *b_f_* and the thickness *t_f_*) and *τ*(*z*) is the value of the interface bond stress at the point of abscissa z.

Moreover, the compatibility condition, schematically depicted in Figure 1, can be written as follows:

(2) $$\varepsilon_{f}=\frac{ds\left(z\right)}{dz},$$

where *ε_f_*(*z*) and *s*(*z*) are the axial strain of the FRP section and the relative interface displacement (hereafter referred to as “slip”) at the generic section of abscissa *z*.

Equations (1) and (2) can be easily linked to each other by introducing the relationship that represents the linear elastic behavior of the FRP strip:

(3) $$\sigma_{f}\left(z\right)=E_{f}\varepsilon_{f}$$

where *E_f_* is the Young modulus of the FRP strip.

Therefore, from Equations (1)–(3), the following differential equation can be obtained:

(4) $$\frac{d^{2}s\left(z\right)}{dz^{2}}+\frac{\tau \left(z\right)}{E_{f}t_{f}}=0.$$

The second assumption listed in Section 2.1 implies that the bond–slip law, namely the relationship between *τ*(*z*) and the corresponding *s*(*z*), can be mathematically written as follows:

(5) $$\{\begin{array}{ll} \tau \left(z\right)=-k_{e}s\left(z\right) & \mathrm{if}\;s(z)\leq s_{e}\\ \tau \left(z\right)=-\tau_{0}e^{-\beta \left(s\left[z\right]-s_{e}\right)} & {\mathrm{if}\;s(z)\text{>}s}_{e} \end{array}$$

where *k_e_* is the stiffness of the pre-peak elastic branch, *τ*_0_ is the maximum bond shear strength, *s_e_* = *τ*_0_*/k_e_* is the corresponding elastic slip value, and β is the parameter that controls the shape of the post-peak exponential branch of the *τ-s* law.

**Figure 2:**
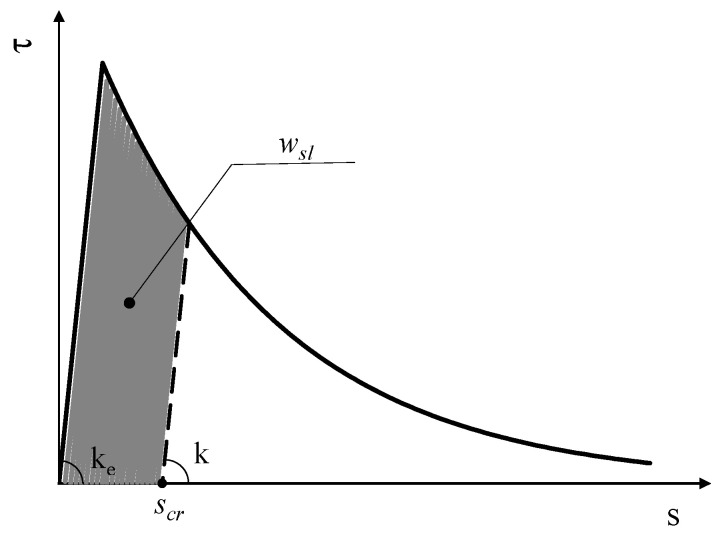
Typical bond–slip law.

Figure 2 shows what a typical bond–slip law assumed in the present study looks like. In addition to the already defined parameters involved in the expression of the bond–slip law (Equation (5)), the dashed line represents the unloading–reloading branch that is relevant in the case of cyclic actions. The area beneath the bond–slip curve has a clear mechanical meaning, as it represents the fracture energy $G_{F}^{II}$, namely the energy needed for opening a fracture (in mode II, under the current assumptions). A closed-form expression can be easily obtained for $G_{F}^{II}$ by integrating the expression of the bond–slip law in Equation (5):

(6) $$G_{F}^{II}=\int_{0}^{\infty }\left|\tau \left(s\right)\right|ds=\frac{k_{e}s_{e}^{2}}{2}\cdot \left(1+\frac{2}{\beta s_{e}}\right)\;.$$

Furthermore, Figure 2 also highlights that the stiffness *k* of the unloading–reloading branch of the bond–slip law can either be equal to *k_e_* or lower than that, as a result of some damage effect depending by the crack-opening slip *s_cr_* and work spent in the fracture process. Further details about the relationship between *k*, *w_sl_*, *s_cr_* and *s* are omitted herein as they were discussed in a previous paper [19].

### 2.3. Numerical Implementation and Experimental Validation

A finite difference numerical procedure was developed and implemented with the aim to solve the equations reported in Section 2.2. The solution scheme as well as the incremental process implemented for simulating the application of cyclic actions (which, in principle, can be implemented either in force or displacement control) are described in detail in a previously published paper [19].

The numerical procedure was validated with respect to the results reported in two different experimental campaigns reported in the literature [13,20]; details about this double validation are reported, respectively, in two previously published works [19,21].

## 3. Parametric Analysis

### 3.1. Parametric Field

A wide parametric analysis can be executed by means of the mechanical model outlined in Section 2 with the aim of investigating the possible correlations between the parameters describing the FRP-to-concrete joint (namely, the bond–slip law, the axial stiffness of the FRP strip and the bond length) and the resulting cyclic response.

It is worth highlighting that, although the bond–slip law expressed by Equation (5) is defined by a minimal set of three parameters (i.e., *s_e_*, *k_e_* and β), in this study, only one parameter is considered for describing the bond length behavior. Specifically, the mechanical properties of the adhesive interface are described by the related value of the fracture energy $G_{F}^{II}$ (hereafter simply denoted as *G_F_* since debonding only develops in mode II, as stated by the second assumption listed in Section 2.1). Conversely, the values of *s_e_* and *k_e_* are set to typical values for FRP-to-concrete joints (namely, *s_e_ =* 0.02 mm and *k_e_ =* 200 N/mm^3^) and kept constant throughout the parametric analysis. This choice is motivated by the fact that the present study focuses on the low-cycle fatigue response, which implies that a significant portion of the FRP-to-concrete interface is expected to respond in the post-elastic branch: this makes the value of *G_F_*, which controls the ultimate capacity of the system, more relevant than the parameters controlling only the elastic branch of the bond–slip law.

That said, the analyses presented in this study are based on assuming the three main parameters and the corresponding fields listed below:

- Fracture energy *G_F_* ranging between 0.6 and 1.0 N/mm;
- Specific axial stiffness *E_f_t_f_* ranging between 60 and 140 kN/mm;
- Bond length *L* ranging between 100 and 300 mm.

### 3.2. Definition of the Related Dependent Parameters

The definition of the cyclic protocol adopted in this study is based on the theoretical value of the strength *F_mon_* of the FRP-to-concrete joints under monotonic action, which can be determined by means of the following well-known equation:

(7) $$F_{mon}=\sqrt{2G_{F}E_{p}t_{p}}\cdot b_{p}.$$

Then, an equal-amplitude force cyclic protocol can be defined by introducing the following expressions of the minimum (*F_min_*) and maximum (*F_max_*) forces *F* that are applied to the loaded end of the FRP-to-concrete joint (Figure 1):

(8) $$F_{min}=\frac{F_{mon}}{2}-\Delta F,\;F_{max}=\frac{F_{mon}}{2}+\Delta F.$$

In the present study, which specifically targets the issue of low-cycle fatigue, the value Δ*F* ranges between 0.30 F_mon_ and 0.45 F_mon_. More specifically, the present parametric study considers four values of Δ*F* (namely, 0.30, 0.35, 0.40 and 0.45).

Therefore, it is worth highlighting that each FRP-to-concrete joints considered in the analyses was subjected to cyclic forces whose values depend on the corresponding strength *F_mon_* it would have attained under monotonic actions.

### 3.3. Typical Behavior of FRP-to-Concrete Joints

**Figure 3:**
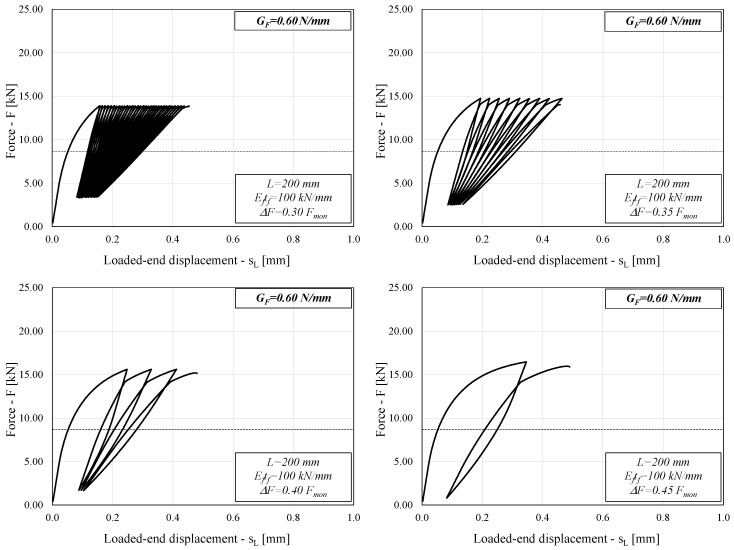
Load–slip curve (L = 200 mm, G_F_ = 0.60 N/mm).

With the aim to show the main features of the cyclic response of FRP-to-concrete adhesive joints, Figure 3 shows four diagrams representing the cyclic response of the system in terms of the applied force F and resulting end slip *s_L_* (Figure 1). Specifically, it refers to the case of G_F_ = 0.60 N/mm and intermediate values of the other two parameters (E_p_t_p_ = 100 kN/mm and L = 200 mm). The four diagrams show the response of the system under cycles of increasing amplitude: as expected, the number of cycles that lead the system to failure is higher in the case of cycles of narrower amplitude, whereas it tends to reduce significantly as the amplitude of cycles becomes wider.

**Figure 4:**
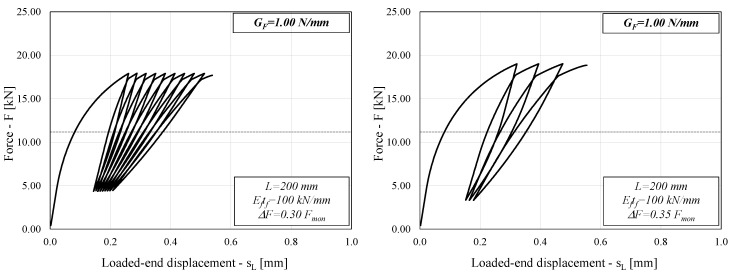

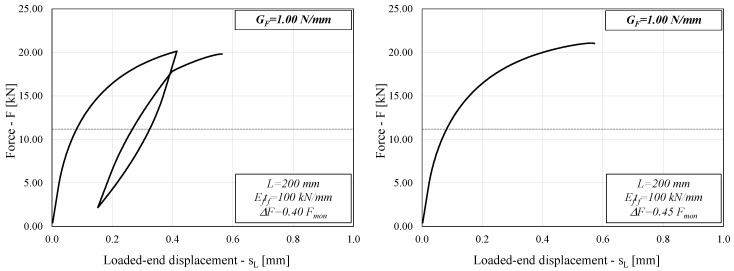
Load–slip curve (L = 200 mm, G_F_ = 1.00 N/mm).

Similar considerations can be drawn from Figure 4, which refers to the case of a higher value of G_F_ (1.00 N/mm) and the same values of the other two relevant parameters.

Comparing Figure 3 and Figure 4 might lead to a seemingly counterintuitive conclusion, as the system with higher *G_F_* (1.00 N/mm in Figure 4) results in a lower number of cycles to failure than the system with lower *G_F_* (0.60 N/mm in Figure 3).

However, it should be noted that each FRP-to-concrete sample analyzed in this study is loaded by different forces that are a function of *G_F_* itself (and, in fact, are as high as the square root of *G_F_*), which results in a different response of the system under consideration. It is reasonable to expect that a higher *G_F_* will lead to a higher number of cycles to failure if the same forces are applied to the system under consideration, but the latter is not the case in the present study.

It is well-known that in the case of monotonic loads, the ultimate capacity *F_mon_* is determined by Equation (7), provided that the bond length *L* is longer than the effective so-called transfer length *L_eff_*, the mechanical meaning of which is widely discussed in the scientific literature [2].

Conversely, in the case of cyclic actions, the results represented in Figure 3 and Figure 4 (as well as preliminary insights presented in a previous study [22]) have shown that the number of cycles to failure is also affected by the ratio *L/L_eff_*. Since *L_eff_* also depends on the bond–slip law properties [4,24], it is easy to understand that the cases analyzed in Figure 3 and Figure 4 have different values of *L/L_eff_* and the reason for the difference observed in terms of number of cycles to failure could be related to this parameter, which was not explicitly defined in the parametric analysis.

The relevance of the *L/L_eff_* ratio in controlling the cyclic behavior of FRP-to-concrete joints will clearly emerge from the in-depth discussion of the results obtained as part of the whole parametric analysis, which is proposed in the following section.

## 4. Discussion

The simulated structural behavior of the systems under consideration can be interpreted as part of the theory of fatigue [23], as the well-known Wöhler curve (also known as the S-N curve) can be obtained by elaborating on the results obtained from the numerical analysis under cyclic actions described in Section 3. Specifically, the amplitude of the cyclic action protocol ∆*F* and the resulting number of cycles to failure *N* can be derived from each one of the analyses run as part of this study.

**Figure 5:**
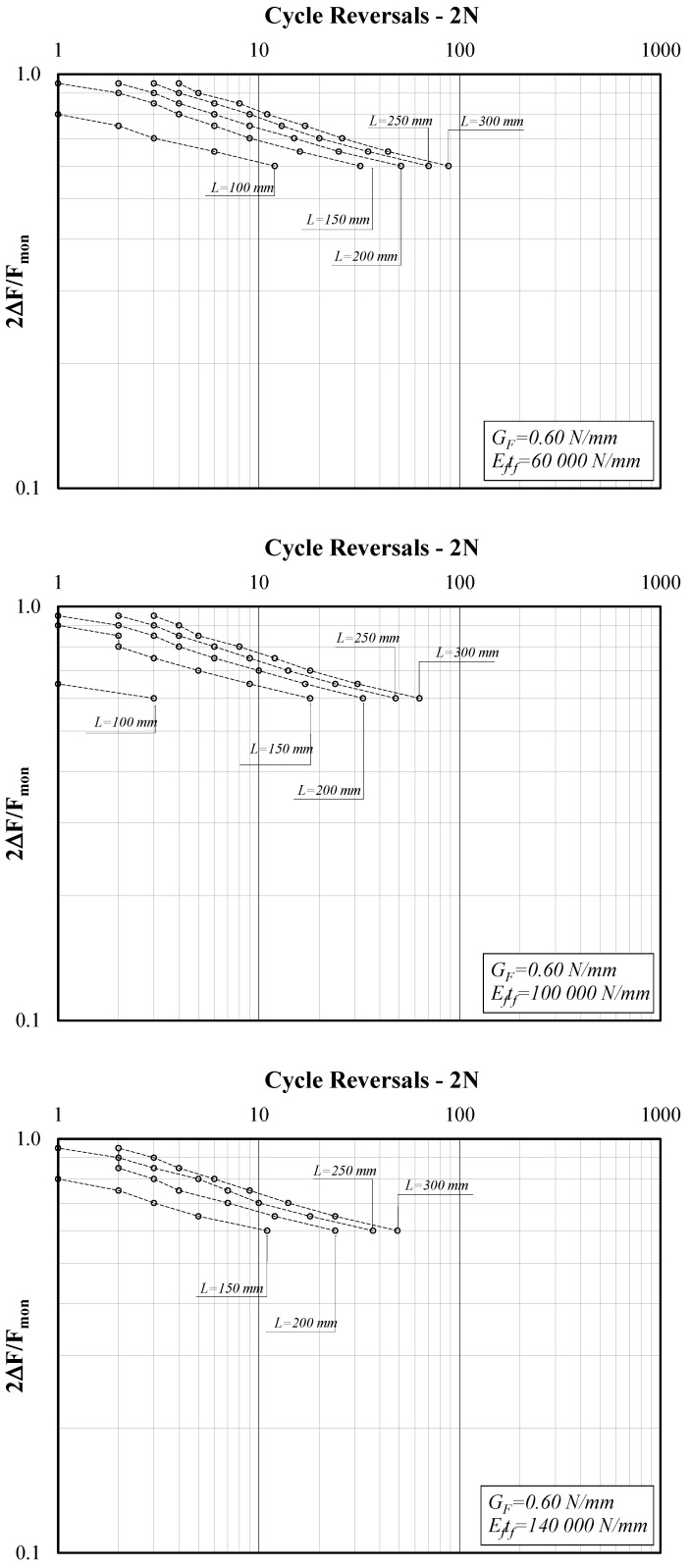
Force amplitude ratio (*2*Δ*F/F_mon_*) vs. cycle reversals at debonding (G_F_ = 0.60 N/mm).

**Figure 6:**
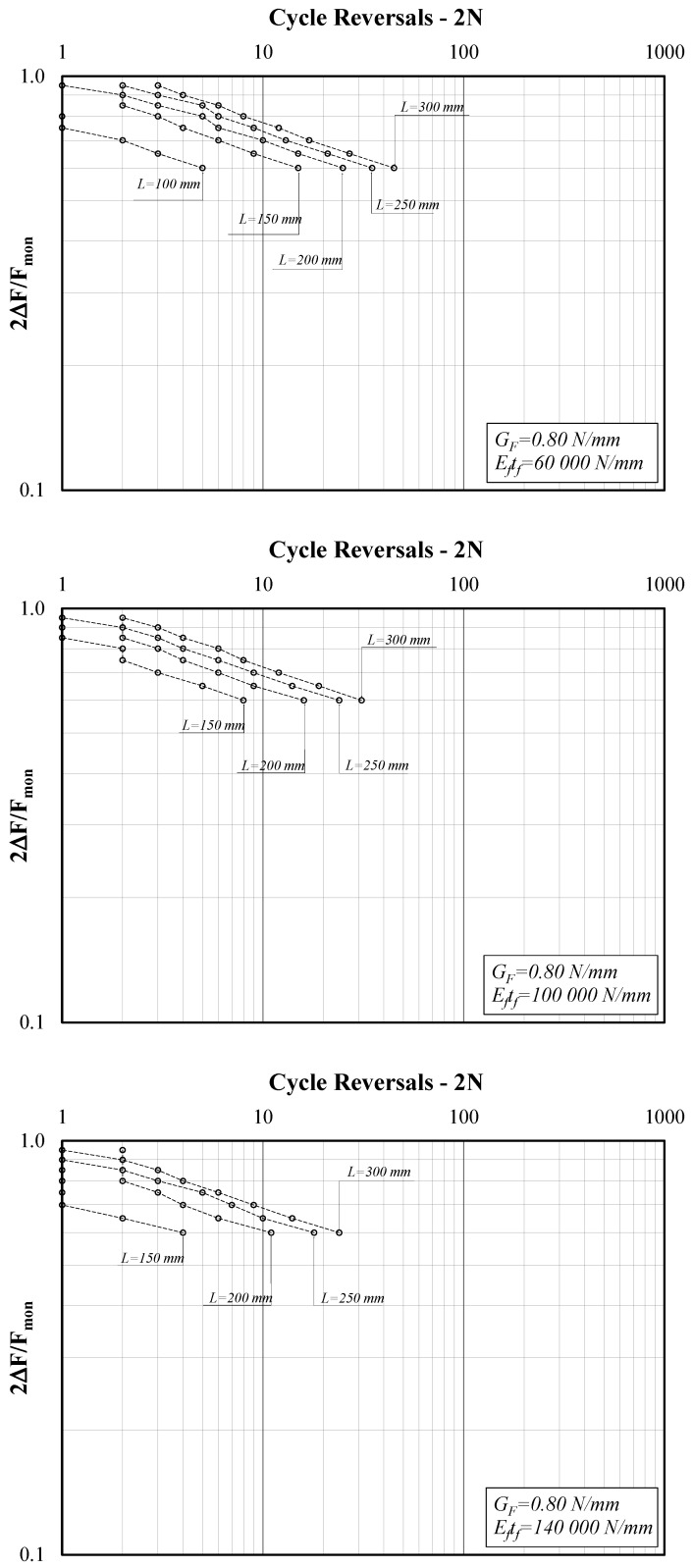
Force amplitude ratio (*2*Δ*F/F_mon_*) vs. cycle reversals at debonding (G_F_ = 0.80 N/mm).

**Figure 7:**
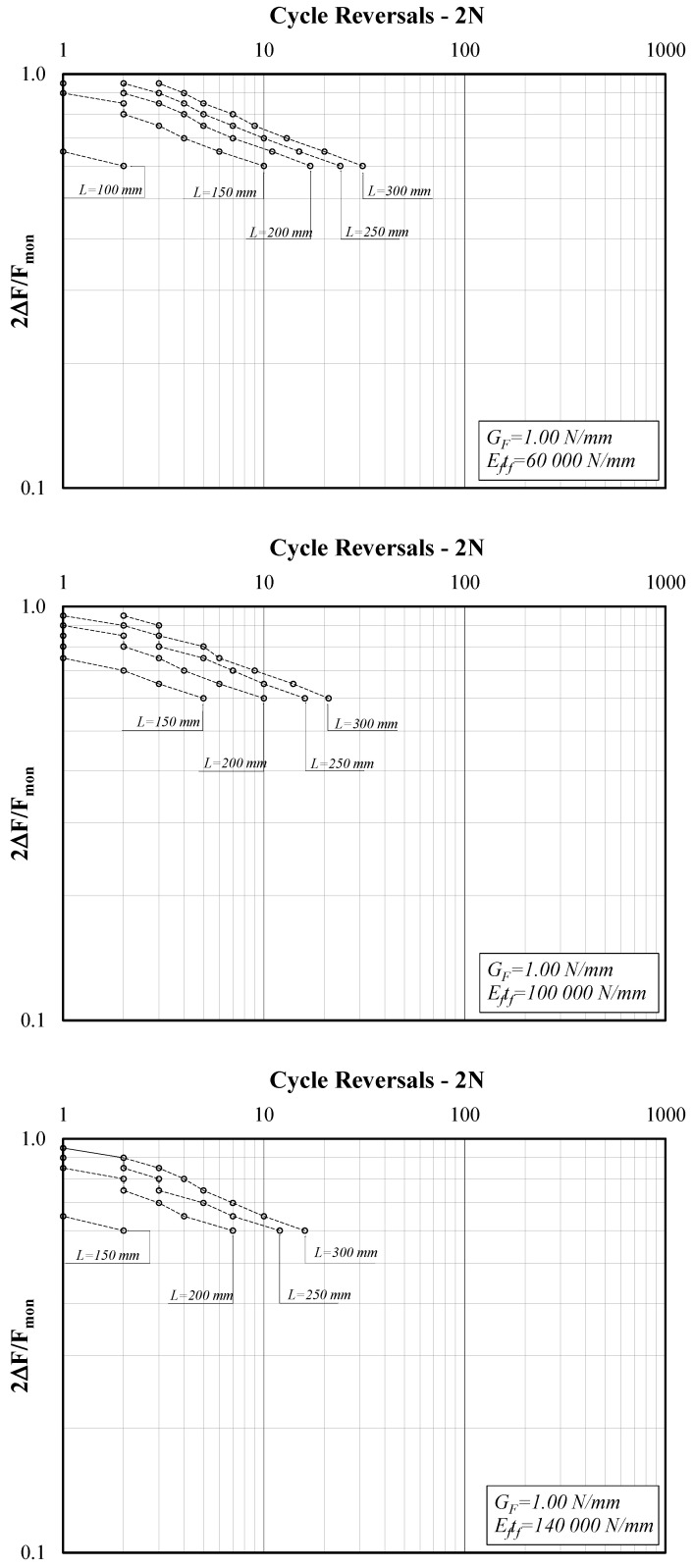
Force amplitude ratio (*2*Δ*F/F_mon_*) vs. cycle reversals at debonding (G_F_ = 1.00 N/mm).

Figure 5 shows three diagrams reporting the force amplitude ratio (*2*∆*F/F_mon_*) on the *y*-axis and the cycle reversals (*2N*) on the *x*-axis, which is one of the possible ways to represent the results of cyclic tests, in view of their interpretation in terms of S-N curves. Specifically, Figure 5 refers to the case of lower *G_F_* (0.60 N/mm) and the three diagrams clearly show the influence of bond length L. Similarly, Figure 6 refers to the case of G_F_ = 0.80 N/mm and Figure 7 to G_F_ = 1.0.

The reported results confirm the “conjecture” put forward at the end of Section 3, as they clearly demonstrate the relevant role played by the bond length *L*. Specifically, if one considers that in the case under consideration, *L* is generally longer than *L_eff_* (the value of which is slightly variable depending on what specific expression is assumed to compute it [13]), the figures highlight that in the case of longer bond lengths, the system can exhibit a higher number of cycles at debonding.

Moreover, the three graphs presented in Figure 5 refer to three different values of the specific axial stiffness *E_f_t_f_* chosen from those within the range of variation of this parameter, as defined in Section 3.1. The comparison among the three graphs clearly shows that the number of cycles to debonding tends to decrease in the case of stiffer strips. In fact, stiffer strips (namely, higher values of *E_f_t_f_*) result in longer *L_eff_* values, and this can be easily understood by keeping in mind the physical meaning of the latter [2].

The three graphs in Figure 6, referring to the case of G_F_ = 0.80 N/mm, confirm the previously highlighted trends of variation in the number of cycles to debonding. Moreover, if compared to Figure 5 (G_F_ = 0.60 N/mm), they show the reduction in number of cycles observed for higher values of *G_F_* (and correspondingly larger applied forces).

Similar considerations arise by analyzing Figure 7, whose three graphs refer to the case of G_F_ = 1.00 N/mm.

**Figure 8:**
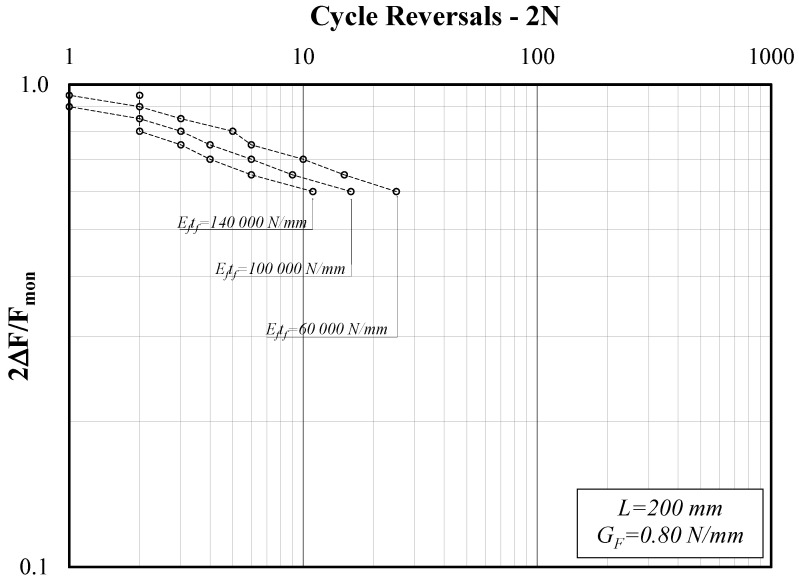
Force amplitude ratio (*2*Δ*F/F_mon_*) vs. cycle reversals at debonding (L = 200, G_F_ = 1.00 N/mm).

Figure 8 focuses on showcasing the role of *E_t_t_f_* in the case of intermediate values assigned to the other two parameters (*G_F_* and *L*), within the definitions of the present parametric study. It clearly confirms that, for the same bond length *L*, stiffer strips (which, among other things, mean longer effective transfer length values *L_eff_*) result in a lower number of cycles at failure.

The graphs plotted in Figure 5, Figure 6, Figure 7 and Figure 8 clearly show that the series of points obtained for each set of values of the triplet (*G_F_*, *E_f_t_f_*, *L*) and the variable values of the amplitude ratio *2*∆*F/F_mon_* are almost aligned in the adopted log-log plane. Thus, they can be represented by the well-known power-expression for relating the amplitude parameter (*2*Δ*F/F_mon_* in the present study) and the corresponding number of cycle reversals *2N*:

(9) $$\frac{2\Delta F}{F_{mon}}=a\cdot {\left(2N\right)}^{b}.$$

where the constants *a* and *b* control the position and slope, respectively, in the aforementioned log-log representation.

Therefore, a couple of values (*a*, *b*) can be derived by interpolating the series of points (*2N*, *2*∆*F/F_mon_*) obtained for each system described by the triplet (*G_F_*, *E_f_t_f_*, *L*) and represented in the aforementioned log-log plane. Moreover, for each system, a conventional value of the effective transfer length *L_eff_* is defined herein as twice the “critical” bond length $\bar{L}$, which was determined in a previously published study where a bilinear bond-slip law was assumed [24]. The present formulation is based on an elastic-exponential bond–slip law, which clearly leads to longer transfer lengths.

**Figure 9:**
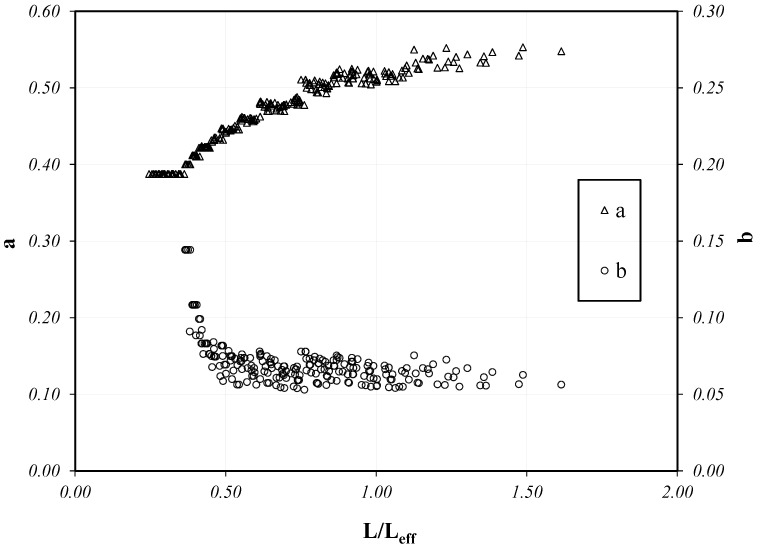
Correlation between *a* and *b* and the *L/L_eff_* ratio.

Figure 9 represents the correlation between the obtained values of both the factor *a* and the exponent *b* (*y*-axis, on the left and on the right, respectively) determined for each one of the systems under consideration and the corresponding *L/L_eff_* ratios.

As can be seen, the *L/L_eff_* ratio plays a significant role in controlling the position and slope of the resulting S-N curves for all the systems analyzed in the present parametric study. Particularly, it is apparent that the variation in both *a* and *b* becomes less pronounced as the value of the *L/L_eff_* ratio grows. In addition, two reference asymptotic values were determined to be in the order of a≈0.50 and b≈0.05 throughout the whole parametric field explored in the present study in terms of G_F_ (60–140 kN/mm) and E_f_t_f_ (60–140 kN/mm).

Conversely, lower values of *a* and higher values of *b* are achieved for bond lengths *L* shorter than the reference transfer length *L_eff_*, which implies leftward-shifted and possibly steeper S-N curves, leading to a shorter “fatigue life” for shorter bond lengths, as clearly shown by the curves represented in Figure 5, Figure 6 and Figure 7.

## 5. Conclusions

This paper further clarifies some aspects of the cyclic response of FRP strips glued to concrete. Starting from a numerical model formulated, implemented and validated in previous work, the results of a parametric analysis were reported and discussed with the aim to understand the influence of the main relevant parameters (e.g., fracture energy of the interface, FRP axial stiffness, and bond length) on the resulting cyclic behavior of the structural joint under consideration, the latter being described in terms of S-N curves, widely adopted in the theory of fatigue.

- As expected, the force amplitude Δ*F* controls the number of cycles leading to debonding in the analyzed systems: the relationship between the amplitude ratio *2*Δ*F/F_mon_* and the number of cycle reversals *2N* is well-described by S-N curves that are close to straight segments in the usually adopted log-log plane;
- Therefore, two parameters (the factor *a* and the exponent *b*) were determined for each series of numerical simulations carried out for given values of *G_F_*, *E_f_t_f_* and *L* and variable values of the ratio *2*∆*F/F_mon_*;
- The results reported clearly show the essential role played by the bond length L, especially in the case of a “short” bond length, that is to say, values of *L* lower than the effective bond length leading to maximum strength under monotonic loads;
- Far from being considered as the final study on this subject, the results give an idea of the actual order of magnitude of the parameters controlling the S-N curves and the correlation between them and the bond length.

It is worth highlighting that the validity of the results is limited to the case of low-cycle fatigue, which is relevant for applications of FRP-to-concrete joints for seismic strengthening of concrete members. Therefore, further validations of the model and parametric analyses are needed to validate the model in the case of low-amplitude (and high number of) cycles, which is the case for members subjected, for instance, to traffic loads.

## Data Availability

Not applicable.

## Supplementary Materials

The Matlab code implemented as part of the present study is available to Readers in Open Access https://doi.org/10.5281/zenodo.5773837.
